# Static study and numerical simulation of the influence of cement distribution in the upper and lower adjacent vertebrae on sandwich vertebrae in osteoporotic patients: Finite element analysis

**DOI:** 10.1002/jsp2.1343

**Published:** 2024-06-21

**Authors:** Shaolong Huang, Xue Wu, Chengqiang Zhou, Xu Zhang, Zhongjian Tang, Xiangyu Qi, Shuai Zhao

**Affiliations:** ^1^ Department of Orthopedics The Affiliated Hospital of Xuzhou Medical University Xuzhou Jiangsu China; ^2^ Graduate school of Xuzhou Medical University Xuzhou Jiangsu China; ^3^ Department of Orthopedics The Second Affiliated Hospital of Xuzhou Medical University Xuzhou Jiangsu China; ^4^ Graduate School of Wenzhou Medical University Wenzhou Zhejiang China; ^5^ Department of Breast Surgery, The First Affiliated Hospital of Wenzhou Medical University Wenzhou, Zhejiang China

**Keywords:** bone cement distribution, finite element analysis, percutaneous vertebroplasty, sandwich vertebra, spinal force analysis

## Abstract

**Objective:**

We analyzed the influence of the location of the upper and lower cement on the sandwich vertebrae (SV) by computer finite element analysis.

**Materials and Methods:**

A finite element model of the spinal segment of T11‐L1 was constructed and 6 mL of cement was built into T11 and L1 simultaneously. According to the various distributions of bone cement at T11 and L1, the following four groups were formed: (i) Group B‐B: bilateral bone cement reinforcement in both T11 and L1 vertebral bodies; (ii) Group L‐B: left unilateral reinforcement in T11 and bilateral reinforcement in L1; (iii) Group L‐R: unilateral cement reinforcement in both T11 and L1 (cross); (iv) Group L‐L: unilateral cement reinforcement in both T11 and L1 (ipsilateral side). The maximum von Mises stress (VMS) and maximum displacement of the SV and intervertebral discs were compared and analyzed.

**Results:**

The maximum VMS of T12 was in the order of size: group B‐B < L‐B < L‐R < L‐L. Group B‐B showed the lowest maximum VMS values for T12: 19.13, 18.86, 25.17, 25.01, 19.24, and 20.08 MPa in six directions of load flexion, extension, left and right lateral bending, and left and right rotation, respectively, while group L‐L was the largest VMS in each group, with the maximum VMS in six directions of 21.55, 21.54, 30.17, 28.33, 19.88, and 25.27 MPa, respectively.

**Conclusion:**

Compared with the uneven distribution of bone cement in the upper and lower adjacent vertebrae (ULAV), the uniform distribution of bone cement in the ULAV reduced and uniformed the stress load on the SV and intervertebral disc. Theoretically, it can lead to the lowest incidence of sandwich vertebral fracture and the slowest rate of intervertebral disc degeneration.

## Graphical abstract

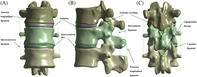

## 1 INTRODUCTION

Osteoporotic vertebral compression fracture (OVCF) is a common fracture type that can lead to persistent pain, kyphotic deformity, and limited mobility, adversely affecting the quality of patients' lives.^1^, ^2^ With the aging population, the incidence and disability rates of OVCF are increasing with about 1.4 million cases every year, resulting in an increase in incidence rate, mortality and medical‐related costs burdening society and families and becoming a major global health problem.^3^, ^4^, ^5^, ^6^ Percutaneous vertebroplasty (PVP) is the percutaneous injection of bone cement into the fractured vertebral body, which can quickly stabilize the fractured vertebral body, strengthen the anterior column, and relieve pain. It is currently the most common minimally invasive method for treating OVCF.^7^, ^8^, ^9^, ^10^, ^11^ PVP is categorized as unilateral PVP and bilateral PVP, which can cause unilateral or bilateral distribution of bone cement in the vertebral body.^12^, ^13^

However, some studies have shown that patients treated with PVP are prone to fractures of adjacent vertebrae that have been reinforced with the incidence of approximately 6.3%–47.5%.^14^, ^15^, ^16^, ^17^ Some researchers hypothesize that bone cement increases the hardness and stiffness of the vertebral body, increasing load on the adjacent vertebral body and leading to fractures.^18^ In addition, the incidence of fractures adjacent to the spine is related to the distribution of cement within the vertebral body.^13^, ^19^, ^20^

Sandwich vertebrae (SV) are normal vertebrae between two cement‐augmented vertebrae and can form when a patient experiences multiple vertebral augmentations.^21^ The SV receives dual load transfer from the upper and lower adjacent vertebrae (ULAV) augmented with bone cement, theoretically increasing its fracture probability than the common adjacent vertebrae.^11^ The emergence of SV is receiving increasing attention, and studies have found that compared to other types of vertebrae, SV is more prone to secondary fractures. Chiu et al. found that the incidence of SV fractures was 21.3%, with approximately half (55.5%) of SV fractures occurring in the first year and almost 90% occurring within the first 2 years. Since the distribution of bone cement significantly impacts the adjacent vertebrae,^22^ exploring the distribution of bone cement that can most reduce load will have a significant impact on preventing secondary fractures in SV.

Therefore, this study established four groups of finite element models to study the biomechanical effects of the distribution of bone cement in the ULAV on SV and the intervertebral disc, providing a biomechanical reference for the clinical treatment of OVCF patients who are likely to form an SV.

## 2 MATERIALS AND METHODS

This study used Mimics 21.0 (Materialize, Belgium), Geomagic 2021 (Geomagic, USA), SolidWorks 2021 (Dassault Systems, USA), and ANSYS 19.0 (ANSYS, USA) software.

### 2.1 The construction of the T11‐L1 finite element model

A healthy male volunteer (70 kg, 175 cm) with no history of spinal trauma or osteoporosis was recruited. The spine was scanned with a 64‐slice spiral CT scanner with a slice thickness and resolution of 0.6 mm and 512 × 512 pixels, respectively. The spinal data after CT scanning were imported into MIMICS software for the three‐dimensional model reconstruction using DICOM format data files.^23^ The reconstructed model was imported into Geomagic Studio 2021 software in STL format. It processed reverse direction as a 3D geometric model to optimize it for surface smoothing, meshing, noise reduction, and fitting surface.^24^

The 3D geometric model, exported as an stp format file, was imported into SolidWorks 2021 software to assemble the vertebral body. The release convex table and segmentation function fabricated the lumbar disc, annulus fibrosus, nucleus pulposus, endplate, and articular cartilage. Linear ligaments were added, which included the anterior longitudinal ligament, posterior longitudinal ligament, interspinous ligament, supraspinous ligament, ligamentum flavum, intertransverse ligament, and capsular ligament. The “Check Interference” command examined the models for interference with each other, with the cortical bone thickness of the vertebral body set at 1.5 mm and the endplate thickness at 0.5 mm.^25^, ^26^, ^27^, ^28^

**FIGURE 1:**
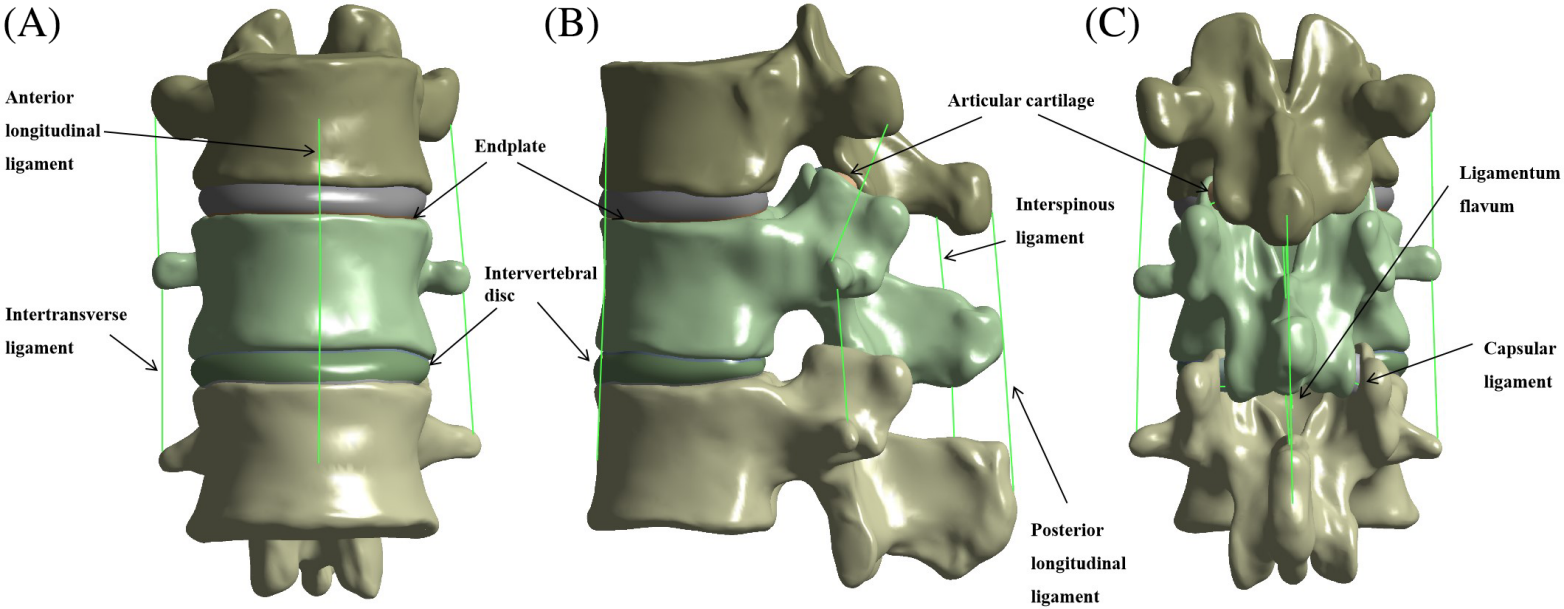
T11‐L1 finite element model. (A) Front view of the T11‐L1 model, (B) left view of the T11‐L1 model, and (C) back view of the T11‐L1 model.

The 3D model in SolidWorks 2021 software was then imported into ANSYS Workbench 19.0 software, and isotropic three‐dimensional entity units were used to mesh the vertebrae, endplates, and intervertebral discs. The spine and intervertebral discs were analyzed as entities with unit types of tetrahedral 10‐node body units, each consisting of a structure with an annulus fibrosus matrix encircling the nucleus pulposus.^28^, ^29^ (Figure 1).

### 2.2 Establishing the bone cement model

This study created two approximately 6 mL cylindrical bone cement (The size of unilateral bone cement is 10.5*10.5*18 mm, and the size of bilateral bone cement is 12*12*14 mm) in SolidWorks 2021 placed them in the appropriate position of the vertebral body (middle or left), and obtained a spinal model implanted with bone cement using the deletion combination.^30^ Finally, the vertebral body model with bone cement under different working conditions was imported into ANSYS Workbench 2021. The mechanical properties of the bone cement were set as linear elasticity, isotropy, and homogeneity, and the interface between the vertebral body and the bone cement was designated as complete binding.^22^, ^30^, ^31^

### 2.3 Experimental group

**FIGURE 2:**
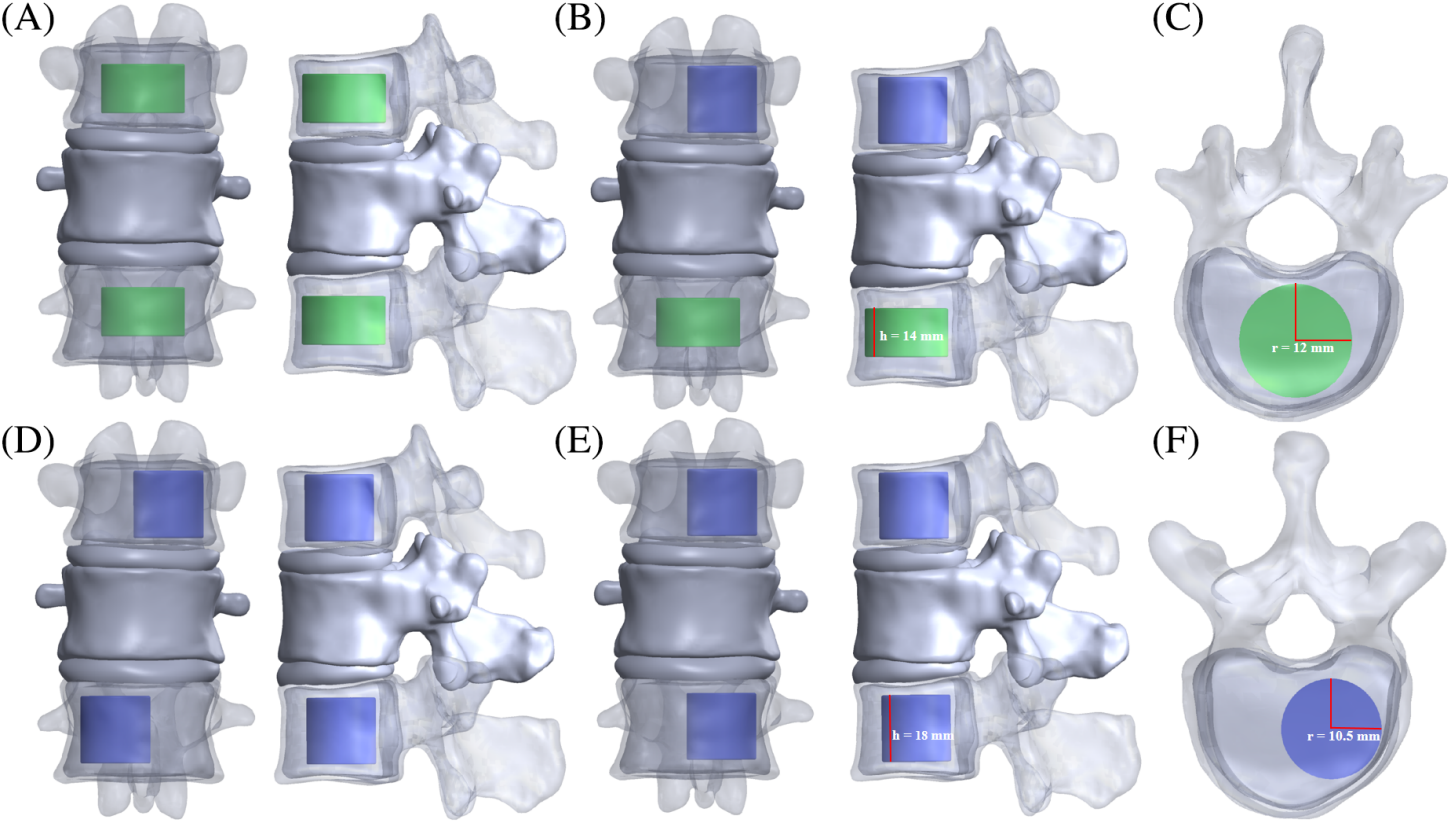
Three‐dimensional models of T11‐L1 for each group. (A) Frontal and lateral view of B‐B group. (B) Frontal and lateral view of L‐B group. (C) Superior view of bilateral distribution of bone cement in T11. (D) Frontal and lateral view of L‐R group. (E) Frontal and lateral view of L‐L group. (F) Superior view of left distribution of bone cement in L1.

The four groups of finite element models were (i) B‐B: bilateral cement augmentation of both T11 and L1; (ii) L‐B: bilateral and left unilateral cement augmentation of L1 and T11 (left), respectively; (iii) L‐R: right unilateral cement augmentation of both T11 and L1; and (iv) L‐L: left unilateral cement augmentation of both T11 and L1 (Figure 2).

### 2.4 Boundary and loading conditions of FE models

**FIGURE 3:**
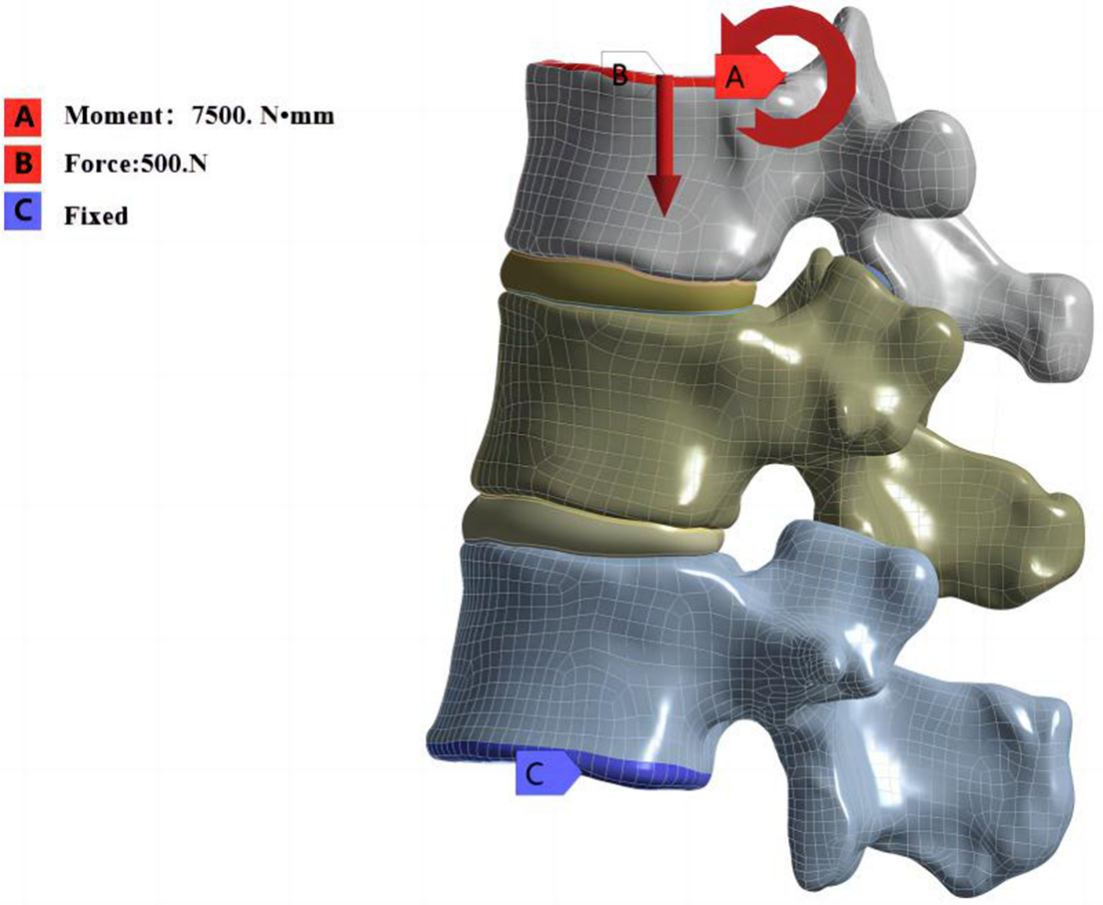
Mechanical model of the spine anterior load.

Restraining the degrees of freedom of all nodes on the lower surface of L1 vertebral body, that is, during loading, no displacement occurred at all nodes on the lower surface of L1 vertebral body. A uniformly distributed downward load of 500 N was added to the upper surface of T11 vertebral body to simulate the upper body pressure under physiological conditions, while a moment of 7.5 N · m was applied on the upper surface of T11 vertebral body to simulate the daily activities of human flexion, extension, left and right lateral bending, and left and right rotation, respectively (Figure 3).

Element types of anterior longitudinal ligament, posterior longitudinal ligament, interspinous ligament, supraspinous ligament, ligamentum flavum, and intertransverse ligament allow tensile deformation without compressive behavior. Facet joint contacts were defined as surface‐to‐surface contact elements combined with a friction coefficient of 0.1,^30^ and contact interfaces of other components were designated as fully bound.^22^ The endplate, cortical bone, cancellous bone, cement, and intervertebral discs (nucleus pulposus and annulus fibrosus) were divided into 2‐mm meshes, while articular cartilage was divided into 0.5‐mm meshes, and the software itself generated meshes and nodes.^28^, ^32^ von Mises stress (VMS) and maximum displacement values were analyzed for T12 vertebrae, T11/T12 and T12/L1 discs. Material properties were defined for each construct (Table 1) and elemental information is shown in Table 2.^26^, ^33^, ^34^, ^35^, ^36^, ^37^, ^38^, ^39^

**TABLE 1:** Material properties information consists of finite elements models.

Parts	Young's Modulus (MPa)	Poisson ratio	Sectional area (mm^2^)
Normal cortical bone	12 000	0.3	–
Osteoporotic cortical bone	8040 (67% of normal)	0.3	–
Normal cancellous bone	132	0.2	–
Osteoporotic Cancellous Bone	34 (67% of normal)	0.2	–
Normal endplate	1000	0.4	–
Osteoporotic endplate	670 (67% of normal)	0.4	–
Cartilage	10	0.4	–
Annulus fibrosus	4.2	0.45	–
Nucleus pulposus	1	0.499	–
Bone cement	3000	0.4	–
Anterior longitudinal ligament	20	0.3	65
Posterior longitudinal ligament	20	0.3	20
Ligamentum flavum	19.5	0.3	40
Supraspinous ligament	15	0.3	30
Interspinous ligament	12	0.3	40
Intertransverse ligament	59	0.3	1.8
Capsular ligament	7.5	0.3	30

**TABLE 2:** Element information consisting of finite elements models.

Group	Node	Unit
Group B‐B	326 827	185 054
Group L‐B	351 304	203 333
Group L‐R	330 120	186 739
Group L‐L	329 881	186 561

## 3 RESULTS

### 3.1 Validation of finite element model of T11‐L1

The current finite element model was validated based on the previously published spine model by applying pure moments and loads under the same conditions to the model, and the resulting range of motion was in good agreement with the literature data (Table 3),^30^, ^40^ demonstrating that the finite element model established in this study is effective and can be used for further analysis.

**TABLE 3:** Range of motion of T11‐T12, T12‐L1, and T11‐L1 compared with previous studies (unit: degree).

	T11‐T12			T12‐L1			T11‐L1		
	Present study	Dai	Liao	Present study	Dai	Liao	Present study	Dai	Liao
Flexion	3.24	3.38	3.00	3.12	3.38	3.30	6.31	6.26	6.60
Extension	2.86	2.84	3.10	3.30	3.74	3.70	6.12	6.18	6.80
Left bending	3.54	3.60	3.30	3.28	3.92	4.00	6.87	6.92	7.30
Right bending	3.68	3.72	3.80	3.34	3.73	3.60	6.82	6.85	6.90
Left rotation	1.82	1.76	2.20	1.24	1.41	1.30	3.23	3.17	3.50
Right rotation	1.88	1.75	2.00	1.29	1.45	1.40	3.27	3.18	3.40

### 3.2 The distributions and magnitudes of the VMS on T12

**FIGURE 4:**
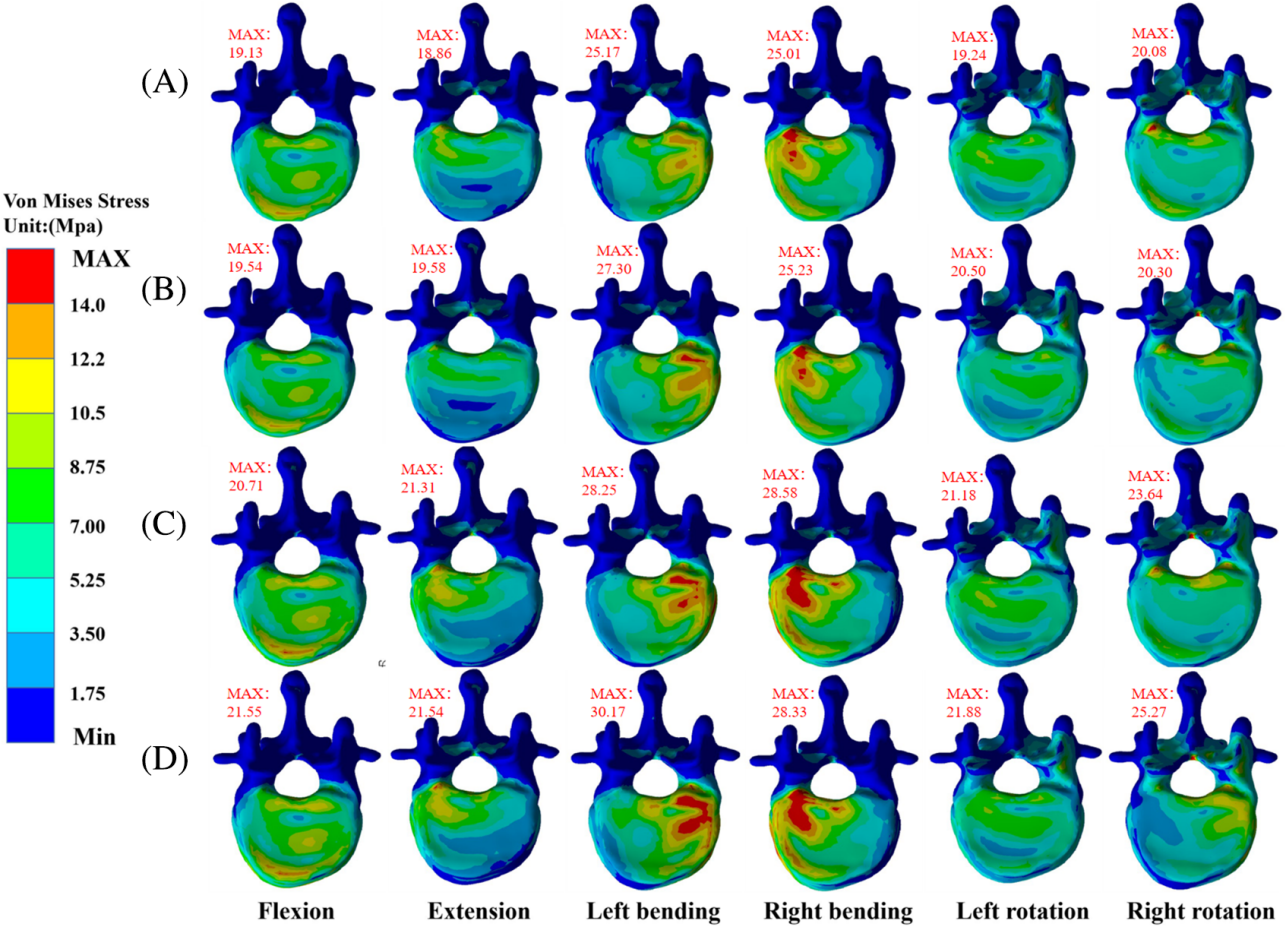
Stress distribution of T12 vertebrae in each group (superior view). (A) B‐B group, (B) group L‐B, (C) group L‐R, and (D) group L‐L.

**FIGURE 5:**
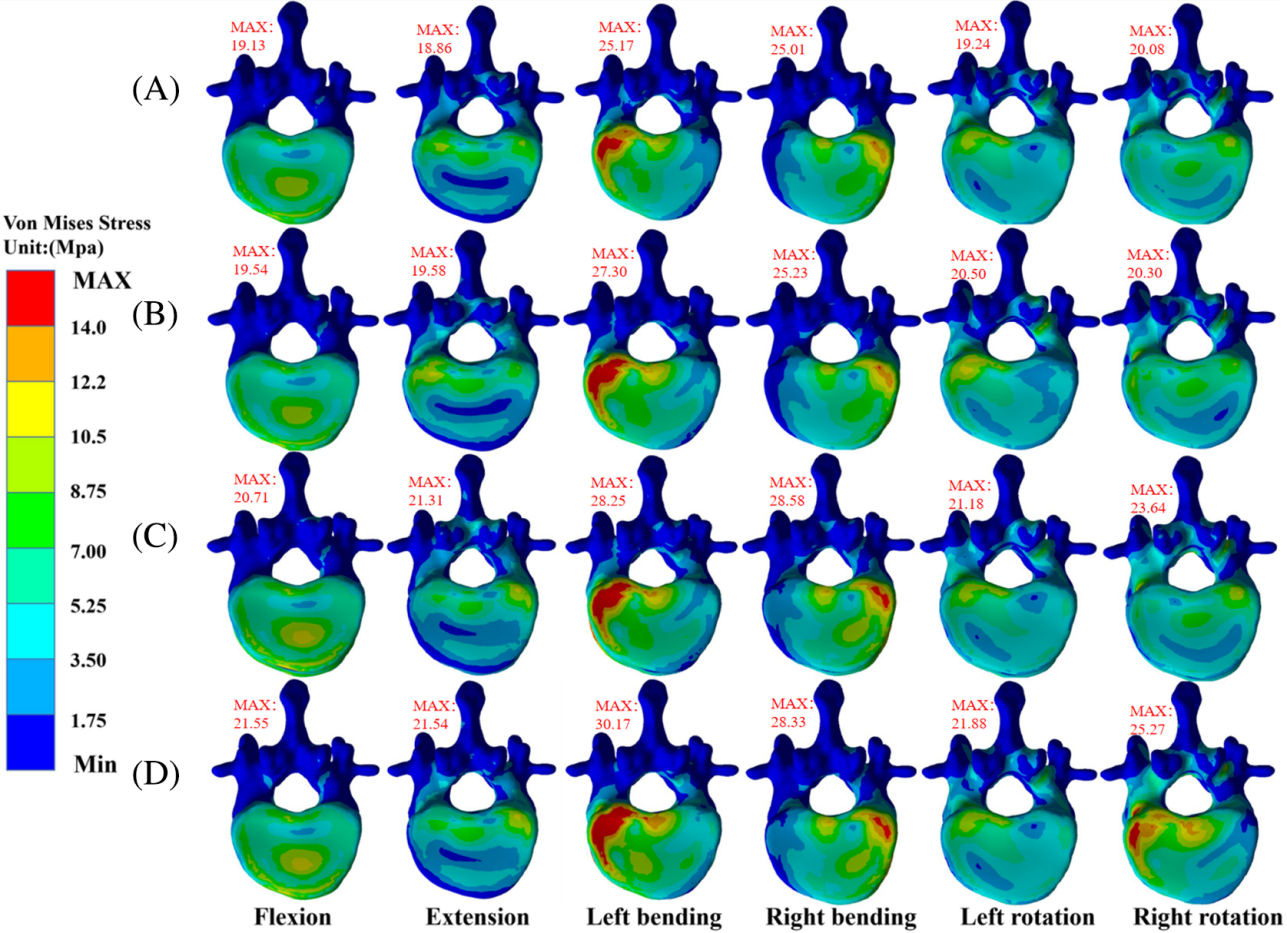
Stress distribution of T12 vertebrae in each group (lower view). (A) Group B‐B, (B) group L‐B, (C) group L‐R, and (D) group L‐L.

The maximum VMS values of T12 vertebrae are in the following order: B‐B < L‐B < L‐R < L‐L group. The VMS stress distribution in Group B‐B is the most uniform, with the maximum VMS values in the six directions of buckling, extension, left and right bending, and left and right rotation being 19.13, 18.86, 25.17, 25.01, 19.24, and 20.08 MPa, respectively. The values of the L‐B group in six directions are 19.54, 19.58, 27.30, 25.23, 20.50, and 20.3 MPa, respectively, and the values of the L‐R group in six directions are 20.71, 21.31, 28.25, 28.58, 19.18, and 23.64, respectively. However, the distribution of VMS in the L‐L group is the most uneven, especially during right‐handed movement, with the maximum VMS in six directions being 21.55, 21.54, 30.17, 28.33, 19.88, and 25.27 MPa, respectively (Figures 4 and 5).

### 3.3 The distributions and magnitudes of the VMS on the intervertebral disc

**FIGURE 6:**
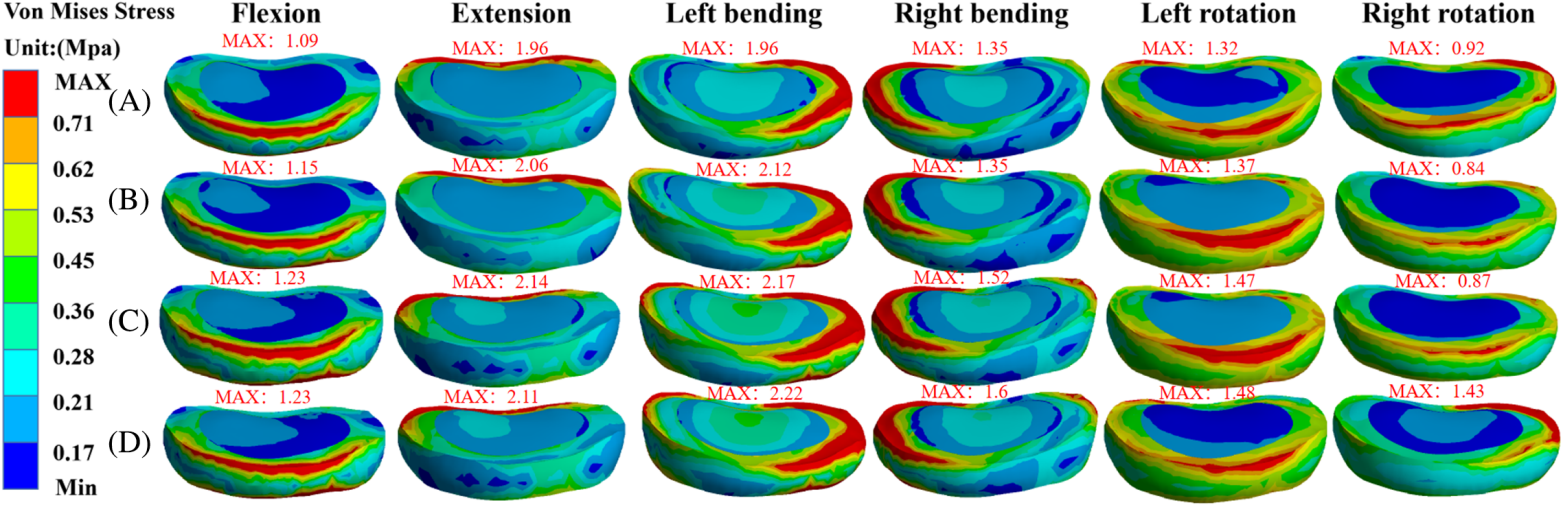
Stress distribution of T11/T12 disc in each group. (A) Group B‐B, (B) group L‐B group, (C) group L‐R, and (D) group L‐L.

**FIGURE 7:**
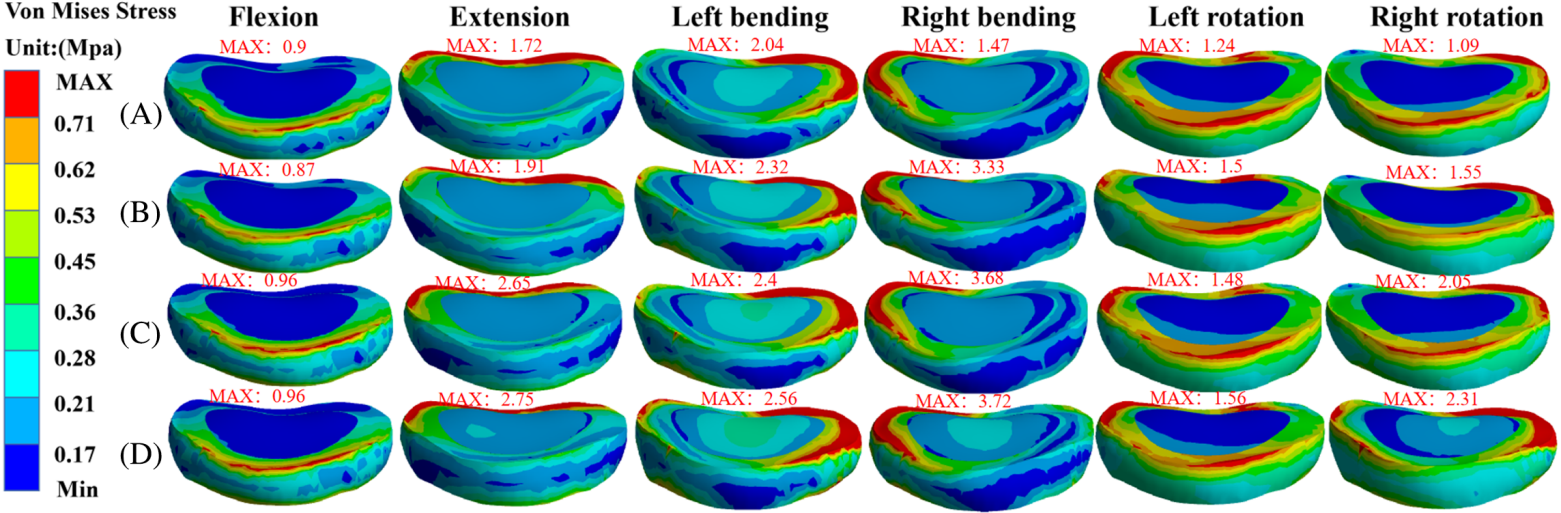
Stress distribution of T12/L1 disc in each group. (A) Group B‐B, (B) group L‐B, (C) group L‐R, and (D) group L‐L.

The stress in the disc tissue was widely distributed over the annulus fibrosus around the disc under six different loading conditions. The stress distribution of the T11/T12 intervertebral disc was concentrated in the load direction during spinal flexion, extension, and left and right lateral bending. The maximum VMS of the T11/T12 disc in group B‐B were 1.09, 1.86, 1.86, 1.35, 1.32, 0.92 MPa in six directions of load flexion, extension, left and right lateral bending, and left and right rotation, respectively. Group L‐B were 1.15, 2.06, 2.12, 1.35, 1.33, and 0.84 MPa in six directions, respectively. The values of L‐R in six directions were 1.23, 2.14, 2.17, 1.51, 1.47, and 0.87 MPa. The group L‐L values in the six directions were 1.23, 2.11, 2.22, 1.61, 1.48, and 1.43 MPa. The maximum VMS in the T12/L1 disc of group B‐B were 0.87, 1.91, 2.32, 3.33, 1.33, and 1.55 MPa in the six directions, respectively, and the values of L‐R in the six directions were 0.96, 2.65, 2.40, 3.68, 1.28, and 2.05 MPa in the six directions, respectively, and the values of L‐L in the six directions were 0.96, 2.75, 2.56, 3.72, 1.36, and 2.31 MPa in the six directions, respectively (Figures 6 and 7).

### 3.4 The maximum displacement of T12

**FIGURE 8:**
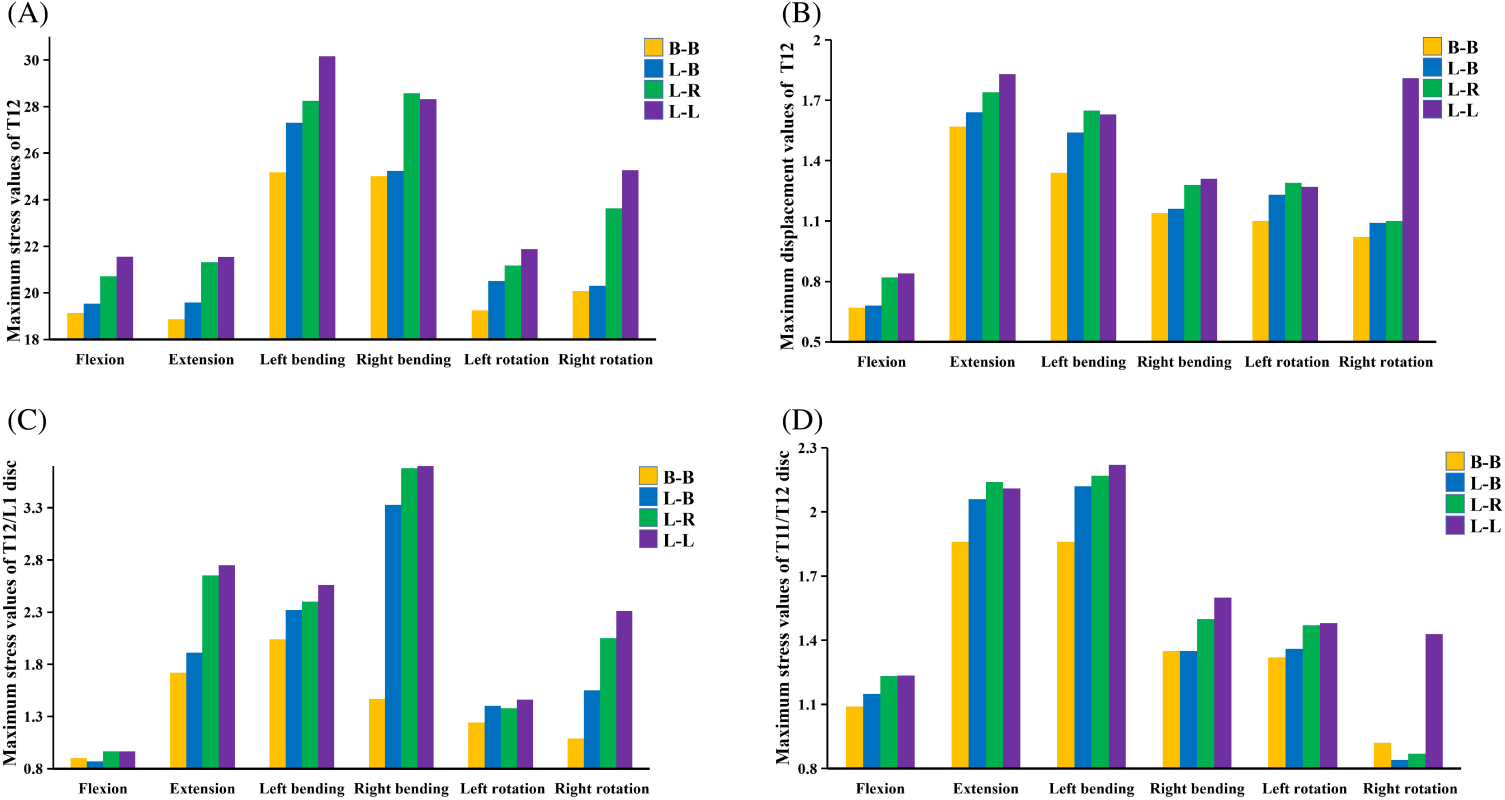
(A) Maximum stress values of T12 in each group, (B) maximum displacement values of T12 in each group, (C) maximum stress values of T11/T12 disc in each group, and (D) maximum stress values of T12/L1 disc in each group.

The maximum displacement of T12 showed a trend of group B‐B < L‐B < L‐R < L‐L in six directions of flexion load, extension, left and right lateral bending, and left and right rotation, respectively. The maximum displacement of group B‐B in six directions was 0.67, 1.57, 1.34, 1.14, 1.1, and 1.02, respectively. The maximum displacement of group L‐B in six directions was 0.68, 1.64, 1.54, 1.16, 1.23, and 1.09, respectively. The values of group L‐R in six directions were 0.82, 1.74, 1.65, 1.28, 1.29, and 1.1, respectively. The values of group L‐L were 0.84, 1.83, 1.63, 1.31, 1.27, and 1.81, respectively. (Figure 8).

## 4 DISCUSSION

Tanigawa et al.^41^ found new vertebral fractures in 33% of 194 PVP patients, who were followed for a mean of 33 months, and 67% of new vertebral fractures occurred adjacent to the reinforced vertebrae. Complications of PKP and PVP include recompression of the operated vertebral body, new fractures of adjacent vertebrae, and degenerative disc disease. A sandwich vertebra is a normal vertebra between two cemented vertebrae, and it theoretically may experience dual loading, resulting in increased stress on itself.^11^, ^42^ The cement distribution significantly influences the adjacent vertebrae,^1^, ^43^ therefore, this study hypothesized that the load on the SV was also related to the cement distribution in the upper and lower vertebrae. This study analyzed the effect of cement distribution on the SV from a biomechanical perspective by the finite element method.

In clinical studies on SV, Jin Liu et al.^44^ reported that the risk of secondary fractures increases with increasing vertebral body type: non‐adjacent vertebrae < normal adjacent vertebrae < SV. Pu Jia et al.^45^ also discovered that sandwich vertebral bodies are more prone to fractures than adjacent vertebral bodies. However, Wang et al.^42^ compared the incidence of Sandwich vertebral fractures (9/42, 21.4%) with traditional adjacent segment fractures (11/71%) in a clinical study, and the difference was not statistically significant (*p* = 0.424). In a study conducted by Ping et al.^11^ on 1347 patients with vertebral compression fractures who underwent bone cement reinforcement, it was found that the incidence of traditional adjacent fractures was 16.4% (196/1194), which was not significantly different from the incidence of vertebral sandwich fractures (*p* = 0.188). We believe that the reason for the divergence of conclusions in these studies may be related to the distribution of intraoperative bone cement and our study shows that the less stress the evenly distributed bone cement causes on the SV, the lower the probability of secondary fractures.

The cement's asymmetric distribution might compromise the physical strength of the non‐augmented side, concentrating stresses received by the adjacent vertebral bodies at certain locations.^46^, ^47^, ^48^ Vertebroplasty increases the collapse of the adjacent trabecular region when the adjacent vertebrae are subjected to fatigue loading.^49^ From the top view of T12, it was observed that the T12 stress in L‐B, L‐R, L‐L groups was more concentrated on the left side of the vertebral body. In contrast, the stress was the smallest on the left side of T12 in group B‐B, indicating that unilateral cement had a higher load on the adjacent vertebral body than bilaterally distributed cement. This observation suggested that symmetrically distributed cement increased the uniformity of the stiffness distribution of the reinforced vertebral body and transmitted a uniform load on the adjacent vertebral body. Dai et al.^30^ found no significant areas of high‐stress concentrations on the anterior and middle vertebral columns reinforced with bilateral cement. Hou et al.^50^ found that the bone cement's symmetrical distribution in the vertebral body provided uniform and stable mechanical support and significantly reduced the incidence of recompression of the injured vertebra. The stress values of the T12 vertebrae under load in all directions were almost in the order of group B‐B < L‐B < L‐R < L‐L. Therefore, the risk of sandwich vertebral fractures is influenced by the distribution of cement in the ULAV: two bilateral distributions < one unilateral, one bilateral distribution < two unilateral (crossed) distributions < two unilateral (ipsilateral) distributions.

The load transmission between the vertebral bodies may be transmitted to the adjacent vertebral bodies along the longitudinal axis of the spine.^51^, ^52^ This study found in the T12 supine view of Group L‐R and L‐L that the right side of the T12 vertebral body in group L‐R was more concentrated than in group L‐L. In contrast, the left side of the vertebral body in group L‐L was more concentrated than in group L‐R, indicating that the bone cement's asymmetric distribution causes stiffness asymmetry of the reinforced vertebral body. The stiffness asymmetry causes unbalanced load transmission, magnifies the stress of the adjacent vertebral body, and increases the risk of new fractures. This study observed that during right rotation, the stress values of the vertebral body and intervertebral disc in group L‐L were concentrated on the left side and were significantly higher than the other groups. In addition, the maximum displacement value of the T12 vertebral body in the L‐L group was 160% higher than that in the L‐B and L‐R, which was due to the fact that the T12 and intervertebral disc in the L‐L group were simultaneously extruded by the left upper and lower bone cement, resulting in higher stress values. Moreover, the bone cement on the upper and lower left sides increased the rotation effect of the right side of the sandwich vertebra, therefore, the rotation action in the opposite direction to the bone cement should be avoided in such patients; otherwise, sandwich vertebral fracture and disc herniation might occur. The maximum displacement value of group L‐R was similar to that of group B‐B and L‐B under the rotation effect because the bone cement on the upper and lower sides was unilaterally distributed, the stiffness of the reinforced vertebral body and transmission load was asymmetric, increasing the stress value in the rotation state.

Baroud et al.^53^ reported that bone cement injected into the vertebral body increased intradiscal pressure due to the transfer of load from the spine between the upper and lower discs. Nagaraja et al.^54^ reported that PKP increased stress in adjacent discs in osteoporotic patients, causing local permanent deformation of adjacent disc spaces. According to Feng et al.,^55^ this observation occurred because prolonged excessive load pressure compromised the nutrient supply to the disc. Dai et al.^30^ found that the peak stress in adjacent discs punctured bilaterally with PVP was less than that punctured unilaterally. Even cement distribution might reduce the incidence of adjacent disc degeneration, which is affected by pressure. The stress values and distributions of T11/T12 and T12/L1 intervertebral discs demonstrated that the bone cement distribution affected the intervertebral disc. The overall stress values of two intervertebral discs gradually increased from group B‐B to L‐L, whereby the T12/L1 intervertebral disc showed more significant performance. Therefore, the uneven distribution of upper and lower bone cement strengthens the stress of the intervertebral disc and accelerates intervertebral disc degeneration. In contrast, the upper and lower ipsilateral bone cement reinforcement threatens the sandwich intervertebral disc. Consequently, the formation of this bone cement distribution should be avoided.

This study found that uniform distribution of bone cement in the ULAV reduced the stress load on the SV and intervertebral disc and decreased the incidence of sandwich vertebral fracture and intervertebral disc degeneration. The upper and lower ipsilateral bone cement reinforcement posed a risk to the sandwich vertebra and intervertebral disc, and this structure should be avoided.

## 5 LIMITATIONS

This study simplified model creation, such as intervertebral discs, endplates, ligaments, and the influence characteristics of paravertebral muscles. In addition, vertical cylinders represented protean cement morphology because the real cement morphology could not be modeled. Liang et al.^56^ found that different shapes of cement simulating PVP produced different stress and displacement values but the same trend; therefore, the same conclusion could be drawn. This study did not cut the injured vertebrae to simulate fractures because this cutting was hypothesized to concentrate angular stresses, causing excessive pseudo stresses. Moreover, this study did not investigate the effect of the amount of upper and lower cement on the sandwich vertebral body or examine whether the sandwich vertebral body was relatively more detrimental than other vertebral bodies. Therefore, more clinical studies and biophysical experiments are needed to inspect these issues.

## AUTHOR CONTRIBUTIONS

Shaolong Huang drafted the manuscript and made the study design. Shaolong Huang and Xue Wu and Chengqiang Zhou completed the finite element analysis. Xu Zhang and Zhongjian Tang collected and analyzed the data. Xiangyu Qi and Shaolong Huang collated and made pictures and tables. Shuai Zhao revised and supervised the manuscript. All authors approved the submitted version.

## CONFLICT OF INTEREST STATEMENT

The authors declare that the research was conducted in the absence of any commercial or financial relationships that could be construed as a potential conflict of interest.

## Data Availability

The datasets used and/or analyzed during the present study are available from the corresponding author upon reasonable request.
